# A comparison of the effects of Chinese non-pharmaceutical therapies for pain control in knee osteoarthritis: A protocol for a systematic review and network meta-analysis

**DOI:** 10.1097/MD.0000000000024501

**Published:** 2021-02-26

**Authors:** Xiaowei Shi, Wenjing Yu, Dongfeng Wang, Ying Zhao, Xiaofeng Deng, Chen Chen, Shufeng Shi

**Affiliations:** a Department of Massage; b Department of Pediatrics, Beijing University of Chinese Medicine Third Affiliated Hospital, Beijing University of Chinese Medicine, Beijing, China.

**Keywords:** acupuncture, knee osteoarthritis, massage, moxibustion, network meta-analysis, non-pharmaceutical therapy

## Abstract

**Background:**

Knee osteoarthritis (KOA) is a chronic degenerative joint disease, leading to pain and functional limitation in the elderly. The non-pharmaceutical therapy is recommended firstly by different guidelines for KOA management strategies. In China, there are various forms of non-pharmaceutical treatments for KOA, which are considered beneficial in relieving KOA pain. However, there is no consensus on which is the optimal non-pharmaceutical regimens. Thus, present network meta-analysis aims to assess the comparative efficacy of available Chinese non-pharmaceutical therapies, especially in pain management.

**Methods:**

PubMed, EMBASE, Cochrane library, Web of Science, China national knowledge infrastructure, VIP, Wan Fang will be systematically searched their inception to April 2020. Randomized controlled trials that compared the effect of non-pharmaceutical therapies on pain control in KOA will be included, including traditional acupuncture, electroacupuncture, warming needle, fired needle, acupuncture followed by moxibustion, moxibustion and massage. The primary outcome was the knee pain levels, and secondary outcome was the comprehensive indicators. Risk of bias assessment of the included studies will be performed according to the Cochrane risk of bias tool. The pairwise and network meta-analysis will be performed by STATA 14.0 and GeMTC softwares.

**Results:**

This study is ongoing and will be submitted to a peer-reviewed journal for publication.

**Conclusion:**

This study will provide a comprehensive evidence on the effects of Chinese non-pharmaceutical therapies for pain control in KOA.

**PROSPERO registration number:**

CRD42018106575

## 1 Introduction

Knee osteoarthritis (KOA) is a chronic degenerative joint disorder among older adults, leading to pain, stiffness, and functional limitation.^[1]^ Cross^[2]^ reported the global prevalence of symptomatic KOA in 2010 was 3.8%. However, the incidence of KOA may be higher in developing countries such as China (8.1%)^[3]^ and India (28.7%).^[4]^ Such high incidence leads to a tremendous individual and socioeconomic burden for all countries,^[5]^ especially in the economically less-developed regions.

Pain caused by KOA can have a significant impact on the progression of the disease, because it can limit the mobility of patients,^[6]^ and cause psychosocial problems such as low self-efficacy and depression.^[7]^ These challenges can lead to a considerable decline in the quality of life. Thus, the current therapeutic approaches for KOA recommended by various guidelines are often divided into 3 main categories: non-pharmacological, pharmacological, and surgical,^[8–11]^ which are primarily aimed at minimizing joint pain and optimizing function.^[12]^

Non-pharmacological approaches were first recommended and considered to be crucial in managing chronic knee pain.^[9]^ With the exception of patient education, exercise and other non-pharmacological therapies in China are similar to those recommended in European and American guidelines. These non-pharmacological strategies include Traditional Chinese Medicine acupuncture, moxibustion, (Mox) and tuina or massage (Mas), which are recommended by the Rheumatology Society^[13]^ and Orthopaedic Society^[14]^ of the Chinese Medical Association.

Acupuncture is probably the most widely recognized complementary and alternative medicine worldwide, with origins in ancient China.^[15]^ The effectiveness of acupuncture in the treatment of pain has been confirmed in cases of musculoskeletal disorders, headaches, and osteoarthritis.^[16]^ However it remains controversial, largely due to a lack of evidence for the underlying mechanism.^[17]^

Mox is an ancient form of thermotherapy widely used in East Asia. It entails the use of direct or indirect burning of dried mugwort on special acupoints. Existing evidence supports the effectiveness of Mox in the treatment of OA pain, and although results have been , they are far from conclusive.^[18,19]^ Since ancient times, Chinese acupuncture methods have led to other combined treatment derivatives, which relieve KOA pain. These derived methods are based on traditional acupuncture (TA) and include electroacupuncture (EA), which combines TA with electrical stimulation, and the warming needle (WN), which combines TA with Mox.

Another non-pharmacological intervention, Mas, is also widely used. In 2012, 15.4 million Americans reportedly used Mas to treat OA.^[20]^ Some randomized controlled trials (RCT) have further verified the efficacy of Mas in KOA.^[21,22]^ Although the effectiveness of these complementary interventions has been reported in many studies, determination of the most effective therapies among non-pharmacological treatments are generally lacking in the field of CAM.

Meta-analysis is a powerful statistical technique, widely accepted as an important tool in evidence-based medicine.^[23]^ Although several direct meta-analyses have been conducted to validate the effectiveness of acupuncture,^[24]^ EA,^[25]^ and Mox^[26]^ in KOA, no relevant review or meta-analysis currently exists among all Chinese non-pharmaceutical therapies. Bayesian network meta-analysis could combine all direct or indirect evidence from different treatment comparisons to facilitate the compilation of a unified, coherent analysis of all trials.^[27,28]^ Therefore, the aim of this network meta-analysis was to assess the comparative efficacy of available Chinese non-pharmaceutical therapies in pain management, with the purpose of providing comprehensive and reliable evidence-based medical evidence in clinical practice.

## 2 Methods

### 2.1 Study registration

The proposed systematic review will be conducted and reported in accordance with the Preferred Reporting Items for Systematic Reviews and Meta-Analyses Protocols statement guidelines.^[29]^

This systematic review and network meta-analysis protocol has been registered on the PROSPERO 2018 (ID: CRD42018106575).

### 2.2 Study eligibility criteria

#### 2.2.1 Type of studies

Only RCTs containing TA, EA, WN, fired needle (FN), acupuncture followed by moxibustion (AM), Mox or Mas against another or against placebo/sham in patients with KOA will be included in this review. Non-randomized studies or patients after knee replacement will be excluded.

#### 2.2.2 Type of participants

Studies that enrolled patients of any age, gender with a clinical diagnosis of KOA will be included.

#### 2.2.3 Type of interventions

We will consider studies evaluating the following treatments: any type of TA, EA, WN, FN, AM, Mox, or Mas used as the sole treatment for KOA, and compared control comparators such as pharmacological treatment or no treatment/placebo, which act as vital arms for the incorporation of indirect evidence in the networks.

#### 2.2.4 Types of outcome measures

##### 2.2.4.1 Primary outcomes

Knee pain levels will be assessed by the visual analogue scale ^[30]^ the Western Ontario and McMaster Universities Osteoarthritis Index (WOMAC) score pain subscale^[31]^ or the 11-point numeric rating scale.^[32]^

##### 2.2.4.2 Secondary outcomes

Comprehensive indicators will be assessed by the WOMAC total scores or the 36-item Short-Form Health Survey for quality of life. In addition, relevant adverse events will be recorded. In addition, relevant adverse events will be recorded.

### 2.3 Search strategy

We will electronically search the following databases from their inception through April 2020: Pubmed, Embase, Cochrane library, Web of science, the China National Knowledge Infrastructure, VIP Information and Wanfang Data. The search approach will use a combination of subject word (keyword) and random words related to KOA, interventions of interest (TA, EA, WN, FN, Mox, AM, Mas, sham/placebo), and RCTs. The search words in the Chinese databases have the same meaning as those used in the English databases. To ensure that the most recent trials will be included, we will also retrieve unpublished protocols and summary results through a search of the clinical trial registry at https://clinicaltrials.gov/. In addition, we will search the previously published reviews and meta-analysis related to KOA using TA, EA, WN, FN, Mox, AM or Mas. There will be no language restrictions in this review.

### 2.4 Identification of studies

The search results from above 7 databases will be imported to ENDNOTE X7 software to data management. After being trained specifically for this study, 2 reviewers (XW Shi and WJ Yu) will screen the articles independently for possible candidates based on the inclusion and exclusion criteria. Any duplicate studies will be removed. After passing the title and abstract screening, the full-text copies of all eligible studies will be downloaded for re-evaluation. A third reviewer (DF Wang or Y Zhao) will consult in cases of disagreement between the first 2 reviewers and rechecked the included studies. Excluded studies and the reasons of exclusion will be recorded.

### 2.5 Data collection

After identification of the target RCTs, 2 independent reviewers (WJ Yu and C Chen) will extract the necessary data using a customised form created by Microsoft Excel 2010 (Microsoft Company, USA, Seattle). One reviewer (XW Shi) will check the accuracy and consistency of all extracted data. The following data will be extracted:

(1) the general information of study such as the authors, year of publication, country, groups, sample size, age, sex;
(2) the detailed treatment information such as diagnostic criteria, parameters of intervention;
(3) pain scores. Other outcome measurements such as WOMAC total scores or 36-item short-form health survey score will be extracted if the study is involved.

### 2.6 Quality of evidence assessment

According to Grading of Recommendations Assessment Development and Evaluation, we will assess the quality of evidence as 4 levels: high quality, moderate quality, low quality and very low quality.^[33]^ In addition, we will use the online guideline development tool to conduct this process.

### 2.7 Risk of bias assessment

The Cochrane Risk of Bias tool,^[34]^ which contains 7 specific domains: sequence generation, allocation concealment, blinding of participants and personnel and other aspects of bias, and the risk of bias of all included RCTs will be assessed with methodological quality as low risk, high risk, or unclear risk of bias. If any domain is scored high/low risk of bias, the study will be considered high/low risk of bias. Two reviewers (Y Zhao and XF Deng) will conduct the quality assessments separately, and a third reviewer (C Chen) will recheck the analyzed data.

### 2.8 Statistical analyses

Firstly, classic pairwise meta-analyses will be synthesized, using the Review Manager Software (version 5.0; the Cochrane Collaboration, Oxford, UK). The results will be reported as the mean difference with corresponding 95% confidence intervals. The Chi-squared test and I^2^ test will be used to assess the heterogeneity across studies.

Secondly, a random-effects Bayesian network meta-analysis will be performed, using the GeMTC software (University Medical Center Groningen, The Netherlands) and the STATA software (version 14.0, StataCorp) for network meta-analysis. Network meta-analysis is a statistical technique used to integrate both direct and indirect evidence for comparisons of interest, which is commonly regarded as its main advantage.^[35]^ Using a full Bayesian evidence network, all indirect comparisons were considered to arrive at a single, integrated, estimate of the effects of all included treatments based on all eligible studies. The Markov chain Monte Carlo method will be applied to calculate the pooled effect sizes. Significant differences were assessed using the upper and lower limits of 95% CIs, without consideration of the value 0. The inconsistency test will be verified using the node-splitting method. According to the quantitative estimation, we will adjust the inclusion of studies and ultimately obtain an ideal network with consistency.

Lastly, network meta-analysis was also able to generate rank probabilities (first, second, and third most effective, etc) for all treatments under evaluation. Probability values will be summarized and reported as the surface under the cumulative ranking (SUCRA).^[36]^ The most effective treatment would have had a SUCRA equal to 100%, while the worst treatment, a SUCRA equal to 0%.^[36,37]^

## 3 Discussion

Typical manifestations of KOA, the most prevalent form of osteoarthritis in the lower limbs, include pain, and limited function.^[38]^ Non-pharmaceutical therapy was first recommended the treatment of KOA by various current guidelines. In China, various forms of non-pharmaceutical therapies can treat KOA pain, including acupuncture (manual acupuncture, WN, and EA), Mox and Mas. Different forms of acupuncture are widely used to pain management of KOA in China, although the recommendations of acupuncture among different guidelines are controversial. Mox, entails the use of dried mugwort (mox) materials, and produces heat to stimulate specific acupoints, with the purpose of treating diseases.^[39]^ It is frequently used in conjunction with acupuncture. It has been well documented that local heat application could reduce pain.^[40,41]^ A recent meta-analysis reported that Mox for KOA was more effective than medication or sham treatments.^[26]^ Mas therapy involves the application of pressure on the joints and muscles to maintain good health or relieve pain. It may be the oldest CAM with evidence of extensive use across most regions worldwide.^[42]^ Although the effectiveness of these complementary interventions has been reported in many studies, determination of the most effective therapies among non-pharmacological treatments are generally lacking in the field of CAM. Thus, this network meta-analysis will provide a detailed summary and analysis of the latest evidence focusing on available Chinese non-pharmaceutical therapies (TA, EA, WN, FN, AM, Mox, or Mas) as well as relevant other treatments for pain management in KOA. Nonetheless, we hope that our findings will assist patients, clinicians and healthcare policy-makers to make a better choice of treatments in KOA, especially the application of non-pharmaceutical therapies in the complementary and alternative medicine.

## Author contributions

**Conceptualization**: Xiaowei Shi, Shufeng Shi.

**Data curation**: Xiaowei Shi, Wenjing Yu, Dongfeng Wang, Ying Zhao.

**Funding acquisition**: Xiaowei Shi.

**Methodology**: Xiaowei Shi, Wenjing Yu.

**Software**: Wenjing Yu, Dongfeng Wang.

**Supervision**: Chen Chen, Xiaofeng Deng.

**Validation**: Shufeng Shi.

**Writing – original draft**: Xiaowei Shi, Wenjing Yu.

**Writing – review & editing**: Dongfeng Wang, Ying Zhao, Shufeng Shi.
